# Tumor Microenvironment Characteristics of Pancreatic Cancer to Determine Prognosis and Immune-Related Gene Signatures

**DOI:** 10.3389/fmolb.2021.645024

**Published:** 2021-06-08

**Authors:** Congjun Zhang, Jun Ding, Xiao Xu, Yangyang Liu, Wei Huang, Liangshan Da, Qiang Ma, Shengyang Chen

**Affiliations:** ^1^ Department of Oncology, First Affiliated Hospital of Anhui Medical University, Hefei, China; ^2^ Department of Hepatopancreatobiliary Surgery, Third Affiliated Hospital of Chongqing Medical University, Chongqing, China; ^3^ Department of Oncology, Xintai People's Hospital, Xintai, China; ^4^ Department of Hepatobiliary and Pancreatic Surgery, The Fifth Affiliated Hospital of Zhengzhou University, Zhengzhou, China

**Keywords:** tumor microenvironment, pancreatic cancer, TMEscore, prognosis, immune checkpoint

## Abstract

**Background:** Pancreatic cancer (PC) is one of the most lethal types of cancer with extremely poor diagnosis and prognosis, and the tumor microenvironment plays a pivotal role during PC progression. Poor prognosis is closely associated with the unsatisfactory results of currently available treatments, which are largely due to the unique pancreatic tumor microenvironment (TME).

**Methods:** In this study, a total of 177 patients with PC from The Cancer Genome Atlas (TCGA) cohort and 65 patients with PC from the GSE62452 cohort in Gene Expression Omnibus (GEO) were included. Based on the proportions of 22 types of infiltrated immune cell subpopulations calculated by cell-type identification by estimating relative subsets of RNA transcripts (CIBERSORT), the TME was classified by K-means clustering and differentially expressed genes (DEGs) were determined. A combination of the elbow method and the gap statistic was used to explore the likely number of distinct clusters in the data. The ConsensusClusterPlus package was utilized to identify radiomics clusters, and the samples were divided into two subtypes.

**Result:** Survival analysis showed that the patients with TMEscore-high phenotype had better prognosis. In addition, the TMEscore-high had better inhibitory effect on the immune checkpoint. A total of 10 miRNAs, 311 DEGs, and 68 methylation sites related to survival were obtained, which could be biomarkers to evaluate the prognosis of patients with pancreatic cancer.

**Conclusions:** Therefore, a comprehensive description of TME characteristics of pancreatic cancer can help explain the response of pancreatic cancer to immunotherapy and provide a new strategy for cancer treatment.

## Introduction

Pancreatic cancer (PC) is one of the most lethal malignant tumors in the world; more than half of pancreatic cancer patients are diagnosed in the terminal stages due to the lack of effective detection methods (Kommalapati et al., 2018; Lee et al., 2019). Furthermore, many cases of PC show resistance to chemotherapy, radiotherapy, and molecular targeted therapy, making the situation more severe (Liu et al., 2019). Poor prognosis and the unsatisfactory results of currently available treatments are largely due to the unique pancreatic TME (Feig et al., 2012).

The tumor microenvironment (TME) is a complex environment in which tumor cells are produced and inhabit (Wu et al., 2019). It consists of a variety of substrates including peripheral blood vessels, immune cells, fibroblasts, inflammatory cells from bone marrow, various signal molecules, and extracellular matrix (Quail and Joyce, 2013). The interaction between tumor microenvironment and tumor cells mediates the immune tolerance of tumor, thus affecting the clinical effect of disease-free treatment (Villar et al., 2015; Savas et al., 2018; Roma-Rodrigues et al., 2019). Drug resistance is a characteristic of tumors, and acquired drug resistance is a formidable challenge to antitumor therapy (Meador and Hata, 2020). Immune checkpoint refers to the intrinsic regulatory mechanism of the immune system, which can maintain self-tolerance and help avoid collateral damage during physiological immune response (Lee et al., 2016; Marcelis et al., 2020). In the TME, cancer cells have selective inhibitory ligands and their receptors, which can regulate T-cell effector function, enhance tumor tolerance, and avoid the eradication by the immune system. Immune checkpoint inhibitors, such as CTLA-4 and PD-1/PD-L1, have attracted worldwide attention in the past several years (Ott et al., 2013). The expression of PD-1 by T cells in the tumor microenvironment can reduce the immune effect mediated by T cells, and the high expression of the PD-1 ligand (PD-L1) in tumor cells can induce tumor cells to tolerate radiotherapy.

In this study, a total of 22 immune cell types and components of cancer-related fibroblasts were estimated based on gene expression profile annotations (Iacobuzio-Donahue, 2012). In the current investigation, we developed a method to quantify the TME penetration pattern (TMEscore) by calculating the TME invasion pattern in 177 patients with PC and systematically correlated the TME phenotype with the genomic and clinicopathological features of pancreatic carcinoma. With the development of technology, the complexity and diversity of TME and its influence on therapeutic response were also deepening. Many studies have proved that it has clinical significance in predicting prognosis and treatment (Zeng et al., 2019; Zhang et al., 2020). TMEscore was a reliable prognostic biomarker and predictor of immune checkpoint inhibitor response in pancreatic cancer.

## Methods

### Data Sources

177 mRNA expressions of spectrum data and clinical data were downloaded from TCGA-PAAD from UCSC Xena (https://xenabrowser.net/datapages/). After removing duplicates and samples with no survival data, there were 177 transcriptome samples used to verify the TMEscore. Of the 177 samples, 176 samples had CNV data and SNP data, and 177 samples had miRNA data and methylation data. From the GEO (https://www.ncbi.nlm.nih.gov/geo/) to download GSE62452 65 samples, the expression of spectrum was used to validate data and clinical data (Table 1).

**TABLE 1:** Pancreatic cancer sample information.

Series accession number	Platform used	No. of input patients	AJCC_Stage	Survival overcome
TCGA-PAAD	Illumina RNAseq	177	I: 21 II: 145 III: 3 IV: 5	OS
GSE62452	[HuGene-1_0-st] affymetrix human gene 1.0 ST array [transcript (gene) version]	65	I: 4 II: 45 III: 10 IV: 6	OS

### Tumor Microenvironment Analysis

We used the immune cell proportion data parsed by CIBERSORT combined with the square sum error in elbow (WSSE group, this method was to find the best cluster number by finding “elbow point”) and the fastest falling point of gap statistic (WK, the K value corresponding to the maximum value of gap) to evaluate the best class number K and used ConsensusClusterPlus R packet to classify to get TMEcluster (k-means, euclidean, and ward. D). In order to make the result more stable, 1000 iterations were selected and combined with survival data to see whether this classification is related to survival.

### Analysis of Correlation Between TMEscore and Prognosis

According to the above TMEcluster results, the clustering results were mapped to RNAseq data, and the limma R package was used to screen for different categories of samples (Ritchie et al., 2015). The screening threshold was *p.* value <0.01 and | log2FC | >1. Category-specific differential genes were selected, the random forest method was used to remove redundant genes to get signature genes, and the functional enrichment of these genes were analyzed to see which pathways were mainly enriched. The genes were divided into two categories by Cox regression model, and the TMEscore was calculated by using the following formula, according to GGI score (Sotiriou et al., 2006).

$${\text{TMEscore}=\sum \log_{2}\left(X+1\right)-\sum \log_{2}\left(Y+1\right)}^{\text{​}}.$$

X is the expression value of the gene set with positive Cox coefficient, and Y is the expression value of the gene set of Cox coefficient

Using the maxstat R package to find the optimal breakpoint for TMEscore, the samples can be divided into two subgroups: TMEscore-high and TMEscore-low. The correlation between these two types of samples and prognosis was further analyzed.

Survival R package was used for survival analysis to analyze the correlation between TMEscore subtypes and clinical outcomes. Survival curves were plotted using survimer R package. Based on Cox regression model, prognosis-related miRNAs and mRNAs were identified, and the survival analysis of these miRNAs and mRNAs was performed.

### Validate the TMEscore Model Using TCGA and GEO Databases

The TMEscore was calculated by using I and II samples in the TCGA database and 65 PC samples in the GSE62452 of GEO, and the best cutin point was found through the maximum selection test. According to the value of TMEscore, the samples were divided into TMEscore-high and TMEscore-low subgroups.

### Analysis of Gene Mutation Characteristics in TCGA-PAAD

A variety of mutation types occur in cancer, including six basic mutation types: C > A, C > G, C > T, T > A, T > C, and T > G. The frequencies of these 96 mutation types were different in different cancers, and the combination of the frequencies of 96 mutation types can be used as a fixed mutation pattern. At present, some mutational signatures were included in the COSMIC database, and some of the mutagenic conditions have been known, for example, the production of signature 4, and signature 29 was related to exposure to smoking. In this article, we used maftools R package (https://bioconductor.org/packages/release/bioc/html/maftools.html) and Somatic Signatures (https://bioconductor.org/packages/release/bioc/html/SomaticSignatures.html) to analyze the mutation of tumor samples, and draw the mutation spectrum and characteristics.

### Analysis of Chromosome Copy Number and Tumor Purity in PAAD

The GISTIC method was used to detect the common copy number variation regions in all samples, including the horizontal copy number variation of chromosome arms and the minimum common region between samples, according to the SNP6 Copy Number segment data. The parameters of the GISTIC method were set as Q ≤ 0.05 as the standard of change significance. When determining the peak interval, the confidence level was 0.95, and when analyzing the horizontal variation of the chromosome arm, the region was larger than the length of the chromosome arm by 0.98 as the standard. The analysis was carried out through the corresponding MutSigCV module of the online analysis tool GenePattern (https://cloud.genepattern.org/gp/pages/index.jsf) developed by Broad Institute.

The purity and ploidy of the tumor were analyzed by ABSOLUTE of R package (https://software.broadinstitute.org/cancer/cga/absolute_download), according to the results of CNV. ABSOLUTE mainly used three submodels—SCNA (CNV data), predesigned cancer karyotype, and somatic mutation frequency to score, and then integrated; the highest score was the optimal model and inferred tumor purity and ploidy.

### Analysis of the Correlation Between TME Models and Gene Expression

The specifically expressed genes in different subsets were identified by the gene expression profile (mRNA and miRNA), and the functional enrichment analysis of specifically expressed genes was carried out to study the biological function differences of different TME model subpopulations.

### Survival Analysis

The DEGs and miRNA were analyzed to see if they were related to survival. Survival R package was used for survival analysis to analyze the correlation between TMEscore subtypes and clinical outcomes. The Kaplan–Meier method was used to analyze the overall survival (OS) stratified by TME score. Statistical significance was defined as two-tailed *p* values <0.05.

### Explore the Relationship Between TMEscore Model and Prognosis of Immune Checkpoint Therapy

Researchers from Harvard developed a TIDE (http://tide.dfci.harvard.edu/) tool to evaluate the clinical efficacy of immunosuppressive therapy. Higher tumor TIDE predictive scores were associated with poor efficacy of immunosuppressive therapy and have a poor prognosis. Because of the five types of tumors with tumor immune dysfunction and rejection characteristics that the researchers were able to calculate, only melanoma had publicly available data on patients treated with anti-PD1 or anti-CTLA4 therapy. Therefore, the prognosis prediction of immune checkpoint therapy for PAAD was completed by TIDE score.

### Statistical Analysis

All statistical analyses were conducted using R (https://www.r-project.org/) or SPSS software (version 25.0), and the *p* values were two-sided. *p* values of less than 0.05 were considered statistically significant.

## Result

### TMEscore Subtypes Were Associated With the Prognosis of PC

**FIGURE 1:**
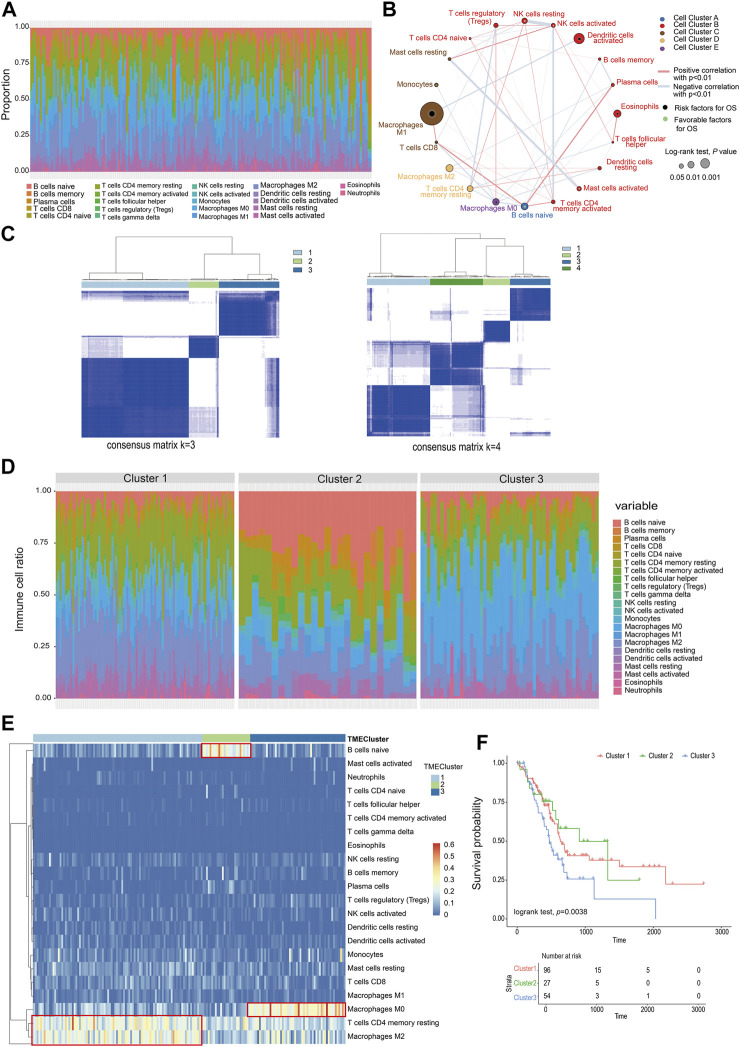
Classification of infiltrating cells and tumor microenvironment. **(A)** The proportion of 22 types of immune cells in the PC sample. **(B)** The relationship between the 22 types of immune cells and their survival (the size of the point represents the correlation between the cell and survival and the thickness of the line represents the correlation between the cells). **(C)** Consensus matrix heat map. **(D)** The proportion of immune cells in different TMEcluster. **(E)** Heat map of different TMEcluster immune cells. **(F)** Different TMEcluster survival analysis.

A total of 22 types of infiltrated immune cell subpopulations were calculated from the RNAseq data of 177 pancreatic cancer samples (Figure 1A). There were correlations between immune cell subpopulations, such as mast cells resting and mast cells activated, NK cells resting, and NK cells activated, and T-cell CD4 memory activated. Through analysis of 22 kinds of immune cells and patient survival data, it was found that macrophage M1 cells were most correlated with prognosis (*p* = 0.000782, 296) (Figure 1B, Supplementary table S1, S2).

Based on the proportions of immune cells, the elbow method and consensus clustering were applied to identify the optimal K value to classify TME patterns, and as a result, three clusters were determined. When *K* = 3, the drop of the elbow curve slows down, which was the best clustering K value (*K* = 3) (Supplementary figure S1, Figure 1C). The result was iterated 1000 times by the ConsensusClusterPlus function (*K* = 1:10) to stabilize the classification and get three clusters (Cluster 1-Cluster 3) (Supplementary table S3) (Monti et al., 2003). The classification of TMEcluster was mapped to the ratio map of immune cells. There was a certain difference in the composition and proportion of immune cells between different TMEclusters (Figure 1D). The differential immune cells in cluster 1 were T-cell CD4 memory resting and macrophage M2, cluster 2 was B cells native, and cluster 3 composed of macrophages M0 (Figure 1E). Combined with the correlation analysis between the final classification results and survival data, it was found that there was a significant difference in the survival time between cluster 1, clusters 2, and clusters 3 (log-rank test, *p* = 0.038) (Figure 1F).

**FIGURE 2:**
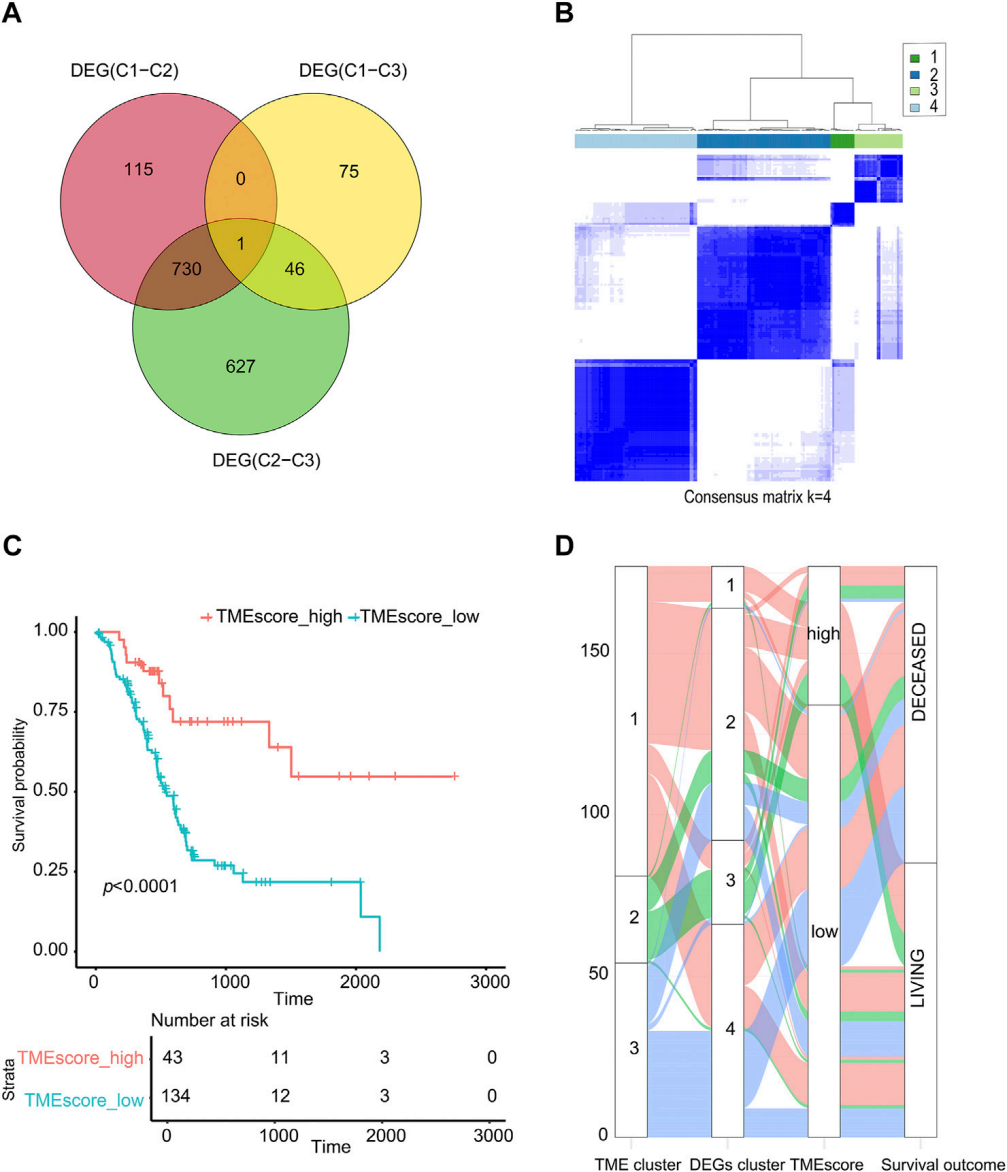
TMEscore value calculation and survival analysis. **(A)** Venn analysis for DEG. **(B)** Cluster analysis of samples based on DEGs. **(C)** The sample was analyzed by TMEscore for survival. **(D)** The alluvial diagram of TMEcluster and the TMEscore group.

According to the TMEcluster classification (*K* = 3), 1594 DEGs were screened by limma R package (*p* < 0.01, | log2fc|>1) (Supplementary table S4). The PC samples were divided into four categories by unsupervised clustering based on the differential genes (Figure 2B, Supplementary figure S2, Supplementary table S5). The Random Forest algorithm was used to de-redundant the DEGs, and in total, 59 signature genes was screened out (Supplementary table S6).

Using the cluster profiler package of R to analyze the functional enrichment of these 59 signature genes, we can see that these significant genes were mainly enriched in the cellular response to extractable stimulus, ras protein signal transmission, and other pathways (Supplementary figure S3, Supplementary table S7). We used the Cox regression model to determine the relationship between these DEGs and the survival of the sample. According to the coefficient value of the gene, the genes were divided into two categories, and the samples were divided into two categories using TMEscore breakpoints. Here, the maxstat R package was used to calculate the best data breakpoints (0.657) so that the samples were divided into TMEscore-high and TMEscore-low subgroups (Supplementary table S8).

As shown in Figure 2, patients had a better prognosis in the TMEscore-high group than those in the TMEscore-low group (*p* < 0.0001). It shown that clustering samples based on immune cell components combined with TMEscore calculation can well-represent the prognosis of samples.

**FIGURE 3:**
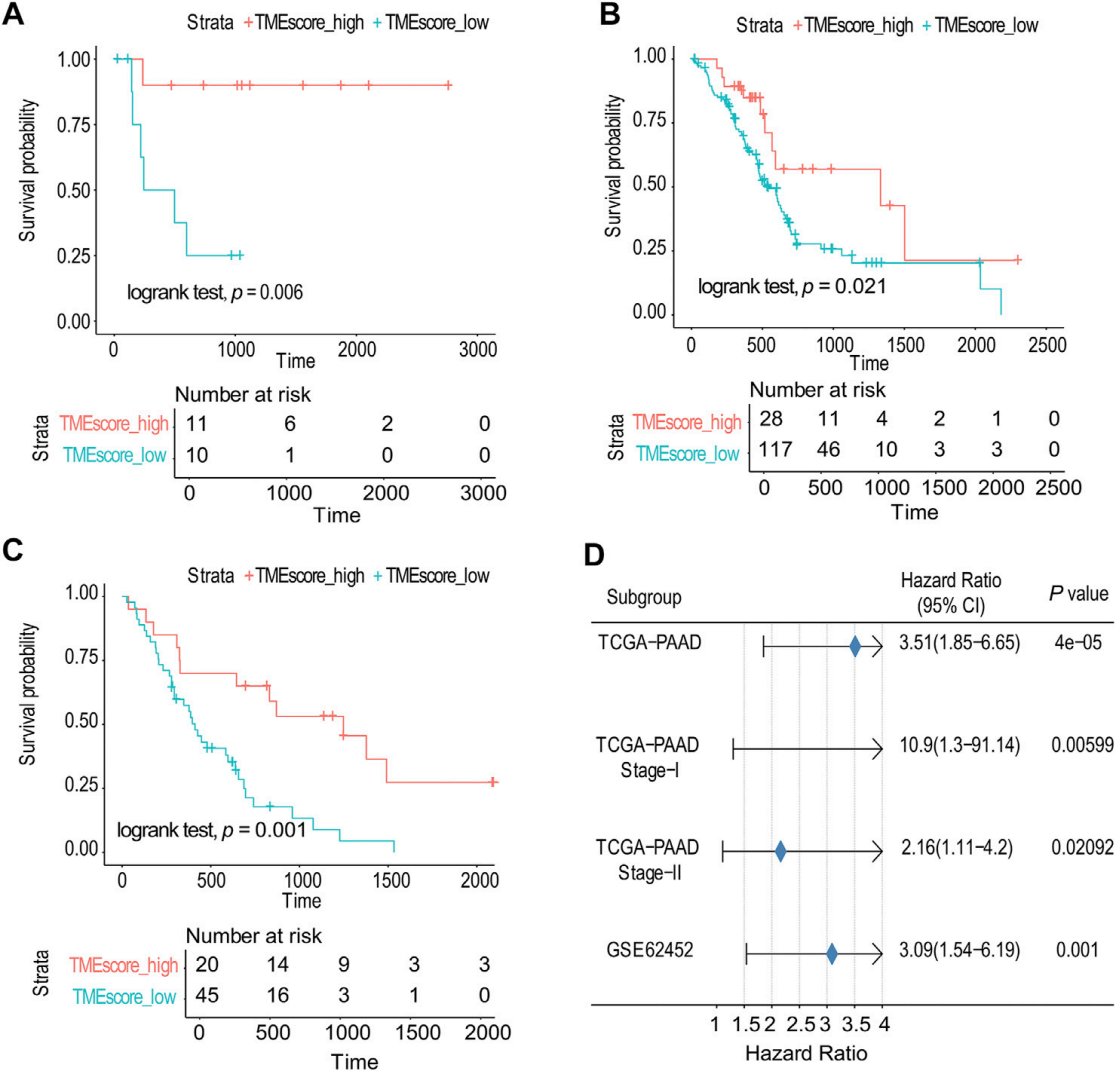
TMEscore model was verified in TCGA and GSE databases. **(A)** Survival analysis of phase I in TCGA-PAAD. **(B)** Survival analysis of phase II in TCGA-PAAD. **(C)** GSE62452 database survival analysis validation. **(D)** Validation and model evaluation were performed using TCGA-PAAD and GSE62452 samples.

Due to the small number of phase III samples (only 3 samples) and phase IV samples (only 5 samples) in TCGA-PAAD database, the TMEscore model can only be applied to TCGA-PAAD phase I, TCGA-PAAD phase II, and GSE62452. In TCGA-PAAD phase I, TCGA-PAAD phase II, and GSE62452 database samples, the survival time of the TMEscore-high group was significantly longer than that of the TMEscore-low group (*p* = 0.006, *p* = 0.02 and *p* = 0.001, correspondingly) (Figures 3A–C; Supplementary table S9). According to the results, patients in the TMEscore-high group had a better prognosis than those in the TMEscore-low group (*p* = 0.00004) (Figure 3D).

### Genetic Characteristics of TMEscore-high and TMEscore-low Subtypes in PC

We made statistical analysis on the mutation data of 176 tumor samples, including the type of mutation annotation, the proportion of different types of base changes, and top 10 mutation genes. In PAAD, missense mutation was the main mutation form, followed by del and ins. Among them, C > T was the most common type of SNP mutation (Supplementary figure S4).

**FIGURE 4:**
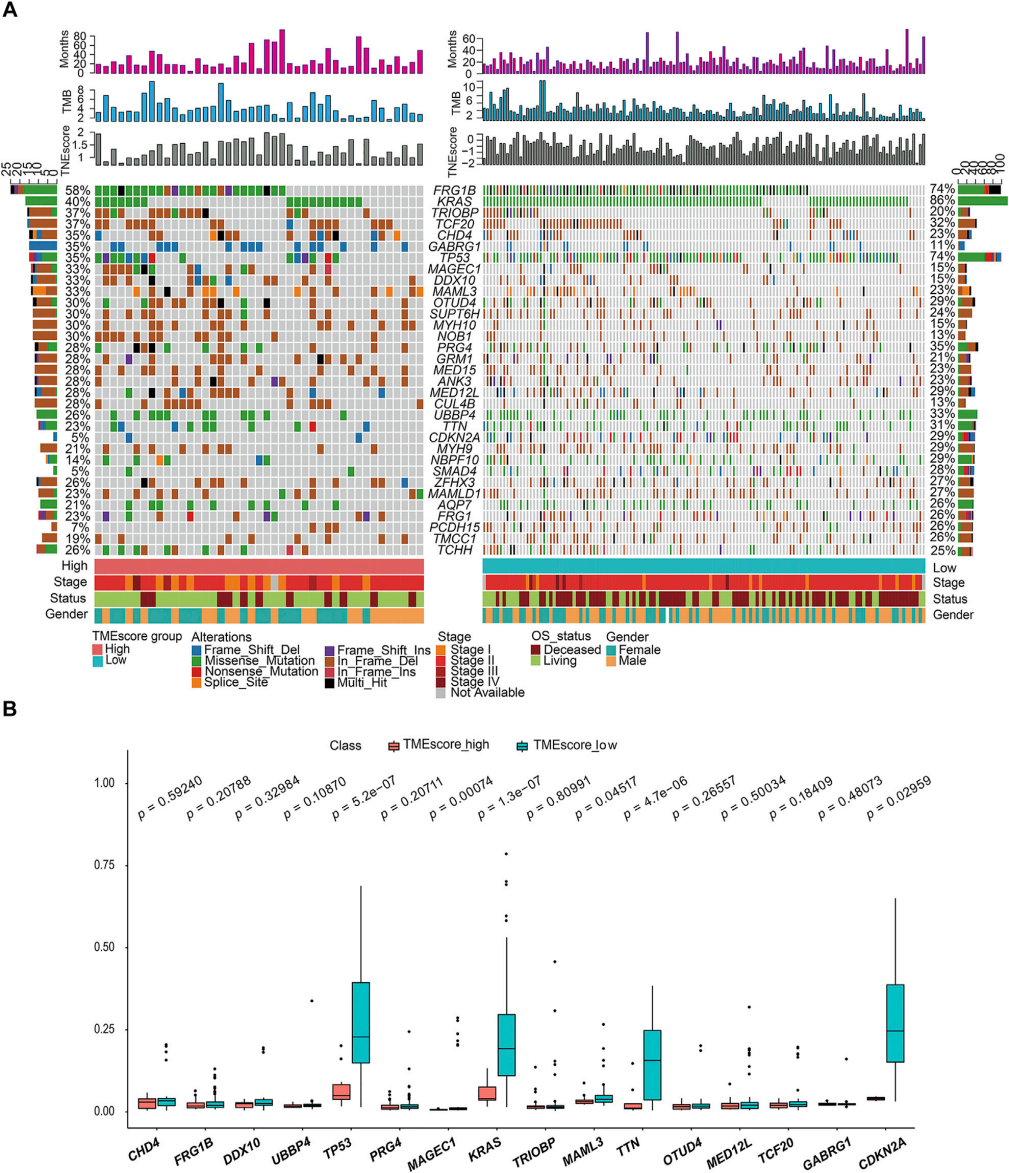
Gene mutation landscape of TMEscore-high and TMEscore-low samples and significant DEGs. **(A)** Distribution and phenotype of common gene mutations in tumor samples of high/low TMEscore groups. **(B)** Analysis of frequency difference of sample gene mutation in TMEscore high/low groups.

According to the union of top 20 mutated genes in the TMEscore-high and TMEscore-low groups of PAAD samples, more than 40% of the samples were mutated in FRG1B and KRAS. Further analysis of the mutation frequency differences of mutated genes in the two groups of high and low TMEscore, it revealed significant differences in the mutated genes of TP53 (*p* = 0.00000053), KRAS (*p* = 0.00000013), TTN9 (*p* = 0.0000047), MAGEC1 (*p* = 0.00047), MAML3 (*p* = 0.04517), and CDKN2A (*p* = 0.02959) (Figure 4).

**FIGURE 5:**
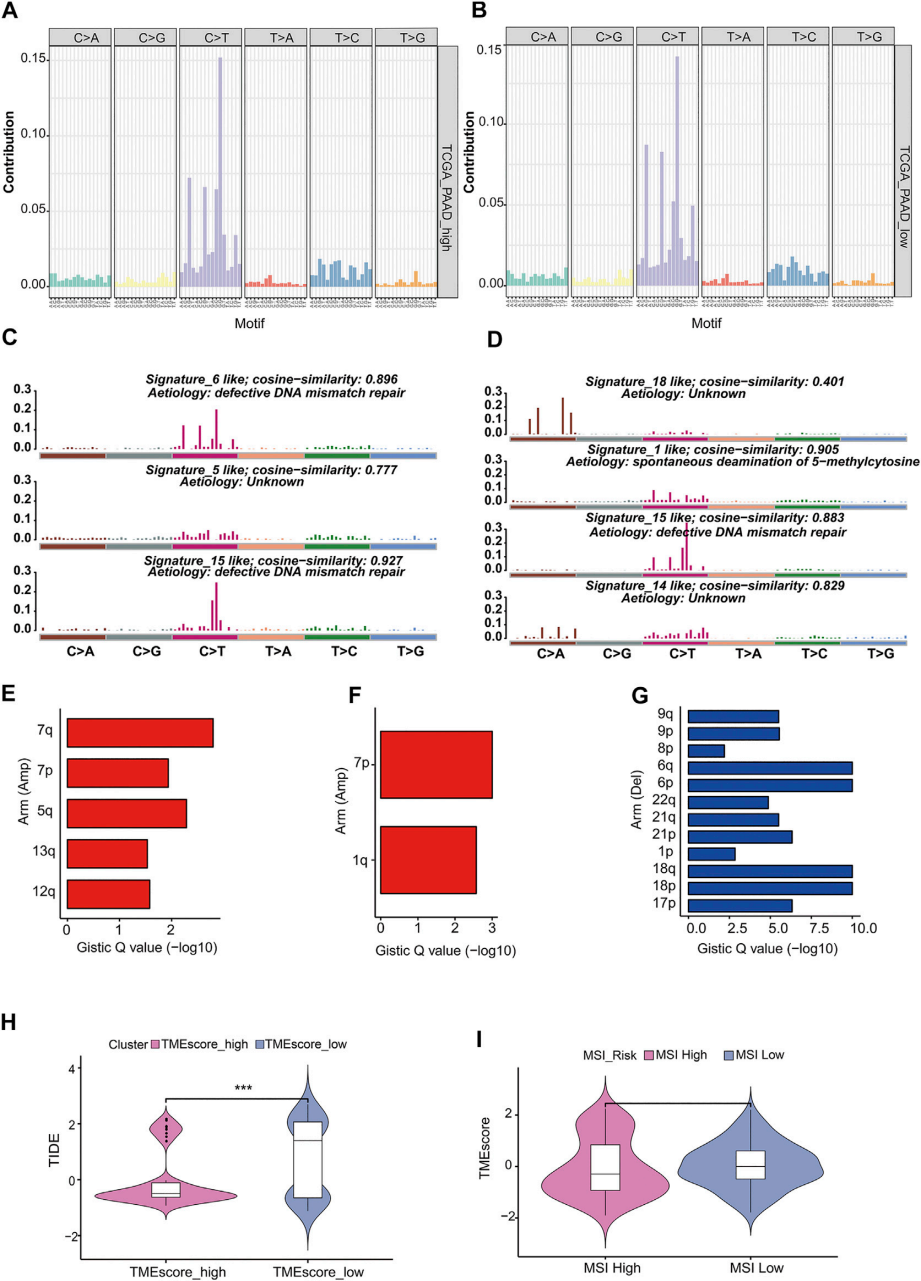
Analysis of mutation characteristics in TMEscore-high and TMEscore-low models. **(A)** Frequency distribution of 96 mutant types of tumors in the high TMEscore group. **(B)** Frequency distribution of 96 mutant types of tumors in the low TMEscore group. **(C)** Mutation signatures of the TMEscore-high group were compared with the similarity of COSMIC dates. **(D)** Mutation signatures of TMEscore-low group were compared with the similarity of COSMIC dates. **(E)** TMEscore-high/low chromosome arm amplification. **(F)** TMEscore-high/low chromosome arm amplification. **(G)** Deletion of chromosome arm level in low TMEscore group. **(H)** Prognosis prediction of immune checkpoint treatment. **(I)** Relationship between MSI risk and TMEscore.

“Mutation fingerprint” of tumor cells and the mutational signatures can be combined with the existing database information to find out which risk factors were mainly responsible for the gene mutation. In order to determine the relationship between the mutation frequency distribution of PAAD tumor samples and the signature included in COSMIC, we decomposed the frequency matrix of 176 samples in row and 96 mutation types in column by nonnegative matrix factorization (NMF) (Figures 5A,B). Three and four distinct somatic mutant signatures were detected in the TMEscore-high and TMEscore-low groups, respectively. Then, we analyzed the similarity between the seven mutation signatures and the signatures collected in COSMIC. We found that TMEscore-high group distinct mutation signatures were mainly related to signature 6, signature 5, and signature 15, while the TMEscore-low group were mainly related to signature 18, signature 1, signature 15, and signature 14. Signature 6 was mainly related to defective DNA mismatch repair, and signature 1 was related to the spontaneous deamination of 5-methylcytosine.

Copy number variation (CNV) was a common form of genomic structural change, which was closely related to the occurrence and deterioration of tumors. GISTIC software was used to analyze the copy number variation of TMEscore-high and TMEscore-low subgroups in PC samples. The amplification of 7p, 7q in the TMEscore-high group was the most significant, and no significant deletion region was found, while the amplification of 7p, 1q and the deletion of 6q, 6p in the TMEscore-low group were the most significant.

Immunosuppressive checkpoint inhibitors were widely used in cancer. By using TIDE to evaluate the clinical effect of immunosuppression treatment of TMEscore-high or TMEscore-low samples, we can see that the TIDE score of the TMEscore-high group was significantly lower than that of the TMEscore-low group (T. test, *p* = 0.0001, Figure 5H, Supplementary table S18). Among them, the higher tumor TIDE prediction score was associated with poorer immunological checkpoint inhibitory efficacy and poorer prognosis.

Studies have shown that patients with MSI-H have better prognosis (PMID: 29531926), and MSI was analyzed in combination with the better prognosis of high TMEscore samples in this analysis (Zeinalian et al., 2018). The MSI score results predicted by TIDE were divided into two groups of MSI-high and MSI-low (R maxstat package prediction best breakpoint was 0.7, MSI-high:111; MSI-low:66). TMEscore between the two groups of MSI-high and -low had no significant difference (*t*-test, *p* = 0.5) (Figure 5I).

### Verification of TMEscore Model in Multiplex of Pancreatic Cancer

**FIGURE 6:**
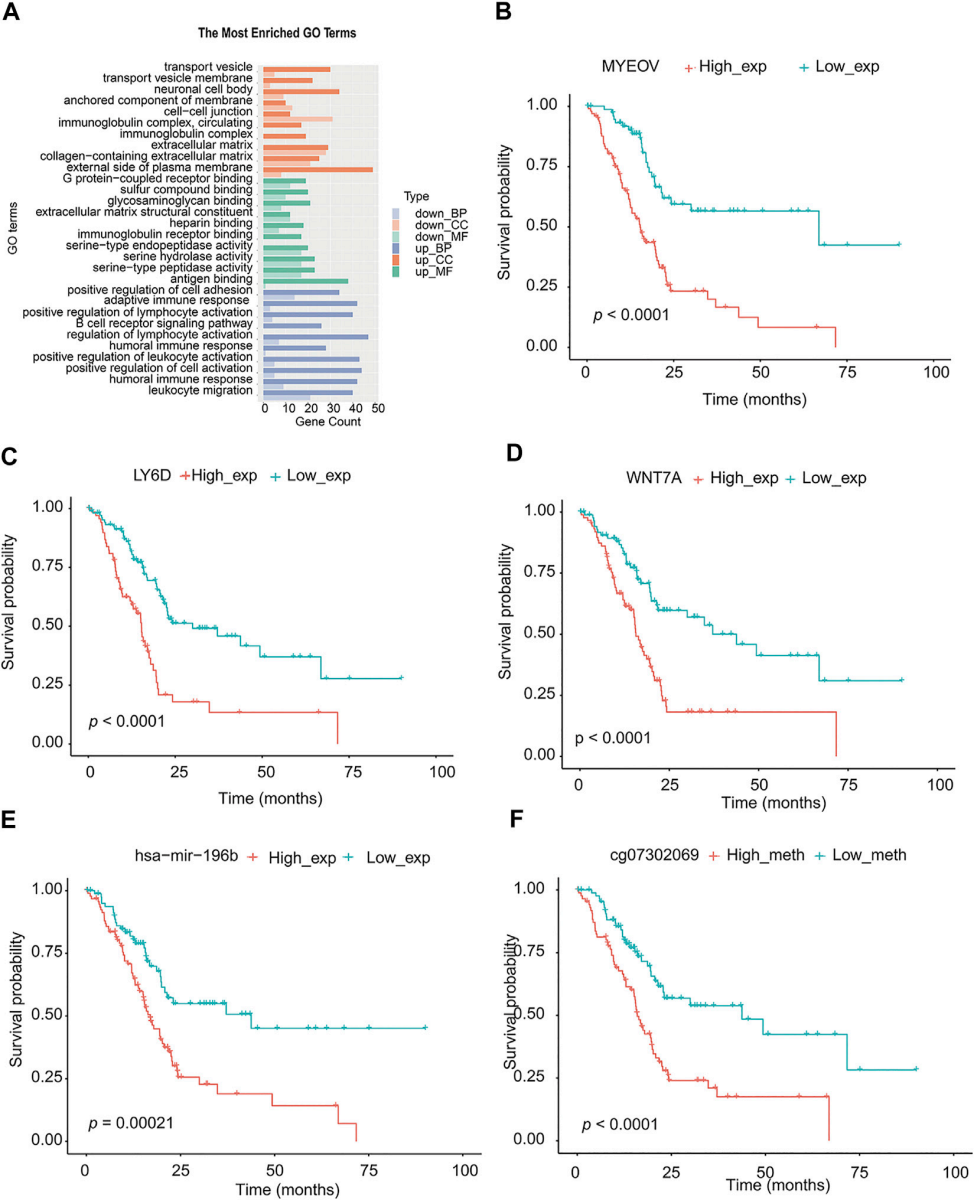
Analysis of signal pathway, partial genes, and prognosis of DEGs in TMEscore-high and TMEscore-low group. **(A)** Enrichment of DEGs by GO. **(B)** Survival analysis of MYEOV gene. **(C)** Survival analysis of LY6D gene. **(D)** Survival analysis of WNT7A gene. **(E)** Survival analysis of hsa-mir-196b. **(F)** Survival analysis of the methylation site of HOXA7.

The miRNA differential expression of TMEscore-high and TMEscore-low groups was analyzed by using the limma R package. The threshold of screening was adj. *p* < 0.05 and the value of | log FC | > 1. A total of 30 miRNAs with differential expression (Supplementary figure S5, Supplementary table S13) were identified. Using the above method, a total of 815 DEGs were obtained (Supplementary figure S6, Supplementary table S14). These DEGs were significantly different between the two clusters (Supplementary figure S7). Using R cluster profiler package to annotate DEGs, we can see that these DEGs were enriched in immune-related pathways such as leukocyte migration and humoral immune response (Figure 6A, Supplementary table S15).

We downloaded the methylation CHIP data of TCGA-PAAD from https://xenabrrowser.net/datapages/and identified the different methylation sites of high/low TMEscore samples (177 samples) with limma R package. A total of 85 significantly different methylation sites (adj. *p* < 0.05) were detected (Supplementary table S16).

According to the above differential miRNAs, DEGs, and differential methylation sites, the samples were divided into high and low expression groups. The log-rank test was used to detect whether differential mRNA, miRNA, and methylation sites were related to survival. According to the threshold of *p* < 0.05, a total of 10 miRNA, 311 DEGs, and 68 methylation sites related to survival were obtained (Supplementary table S17). We selected the top three genes with the highest significance from the mRNA, and the most significant microRNA and methylation sites for the survival diagram (Figure 6 B, C, D, E, F). The differentially expressed mRNA, miRNA, and methylation sites can be used as interesting biomarkers to evaluate the prognosis of patients with pancreatic cancer.

## Discussion

The TMEscore index developed according to the TCGA-PAAD database was a new tool for the comprehensive evaluation of TME. Our results suggested that the assessment of the immune checkpoint through the TME signaling model provides a strong predictor of survival in patients with PC. Macrophage (M1) activates inflammation by activating inflammatory cytokines and reactive oxygen species (Landis et al., 2018). In PC, the relationship between macrophage M1 and prognosis was found to be the closest. At the same time, the TMEscore model was used to predict the prognostic effect, and the prognosis of stage I patients was more significant than that of stage II patients (stage I, *p* = 0.006; stage II, *p* = 0.02). In the previous results, the TMEscore-high group had a better prognosis than the TMEscore-low group. Corresponding to the result of TIDE evaluation, patients in the TMEscore-high group have a good prognosis and the effect of immunosuppression treatment.

Microsatellite instability (MSI) has been proved to be related to the efficacy of immunotherapy (van Velzen et al., 2020). Predicting whether advanced cancer was suitable for immunosuppressive checkpoint inhibitors has almost become a factor that every oncologist must consider, and MSI is one of the important indicators (Pietrantonio et al., 2020). In this study, we did not find a significant difference in MSI risk between the TMEscore-high and TMEscore-low subgroups (Figure 5I).

Using the TMEscore model, we found a large number of potentially important molecular targets in the RNAseq, methylation, and miRNA database of PC. We selected the most differential genes or methylation sites between TMEscore-high and TMEscore-low groups for prognostic analysis. By partially controlling the proliferation of MMC, MYEOV gene expression was used as a prognostic factor in patients with multiple myeloma (Moreaux et al., 2010). In non–small-cell lung cancer, upregulation of the MYEOV transcript was associated with poor prognosis of the disease (Fang et al., 2019). LY6D was a drug-resistant marker gene and therapeutic target for laryngeal squamous cell carcinoma. In addition, the expression of LY6D was associated with pathological T and clinical staging as well as cervical lymph node metastasis (Wang et al., 2020). In cancers with high LY6K expression that were difficult to treat, such as cervical, pancreatic, ovarian, head and neck, lung, stomach, and triple-negative breast cancer, inhibition of LY6K expression through small-molecule binding can be used to inhibit the growth of cancer cells (Newell et al., 2020). Overexpression of the HOXA7 gene can increase the proliferation of liver cancer, breast cancer, and granulosa cells, which was expected to become an important molecular target for the diagnosis and treatment of liver cancer (Zhang et al., 2010; Zhang et al., 2013; Li et al., 2015).

The TMEscore model can be effectively used to predict the prognosis of patients with PC and the prognosis of immune checkpoint therapy. Since not all patients with high TME can get greater benefits from immunotherapy, more clinical factors should be included in the predictive model to improve the accuracy of prediction. In the current study, this comprehensive evaluation of cellular, molecular, and genetic factors associated with TME infiltration patterns helps explain the response of PC to immunotherapy and may provide new strategies for the treatment of pancreatic cancer.

## Data Availability

The datasets presented in this study can be found in online repositories. The names of the repository/repositories and accession number(s) can be found in the article/Supplementary Material.
