# Supplementary material for: Tumor Microenvironment Characteristics of Pancreatic Cancer to Determine Prognosis and Immune-Related Gene Signatures
Source: Front Mol Biosci. 2021 Jun 8;8:645024. doi: 10.3389/fmolb.2021.645024 (PMC8217872; doi:10.3389/fmolb.2021.645024)
Supplement: Supplementary file 1 [file Presentation1.PPTX]

## Slide 1
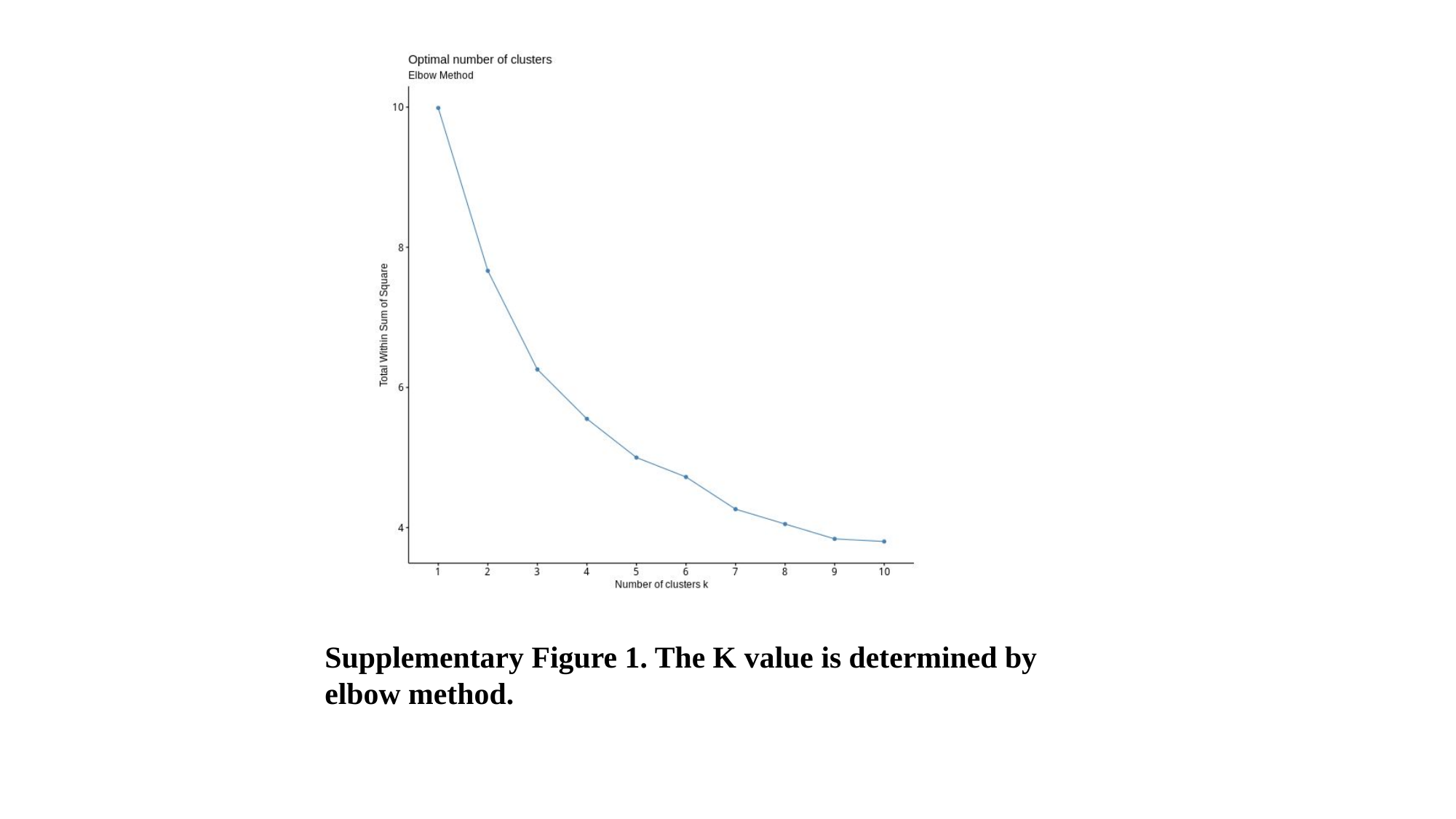

Supplementary Figure 1. The K value is determined by elbow method.

## Slide 2
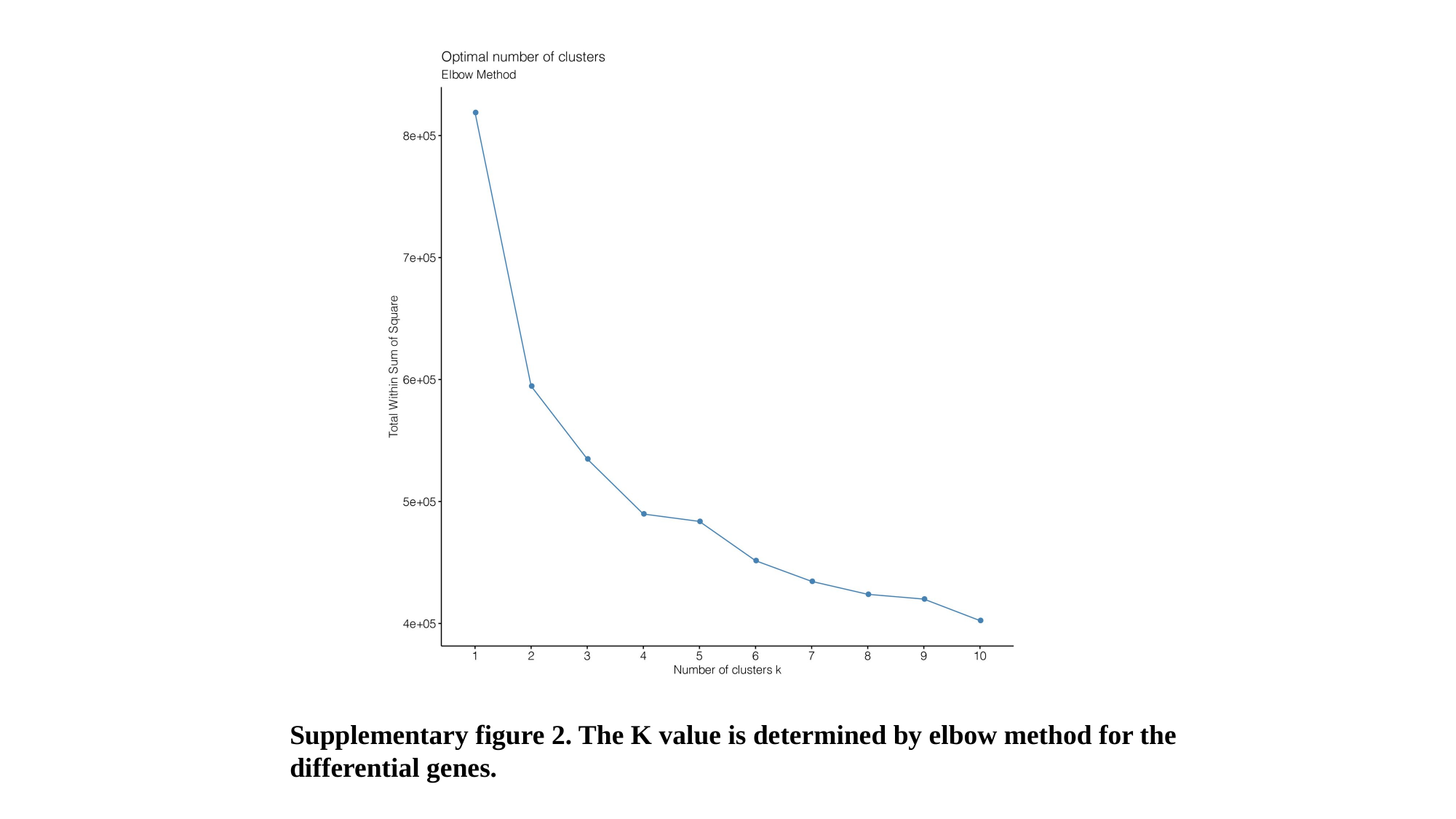

Supplementary figure 2. The K value is determined by elbow method for the differential genes.

## Slide 3
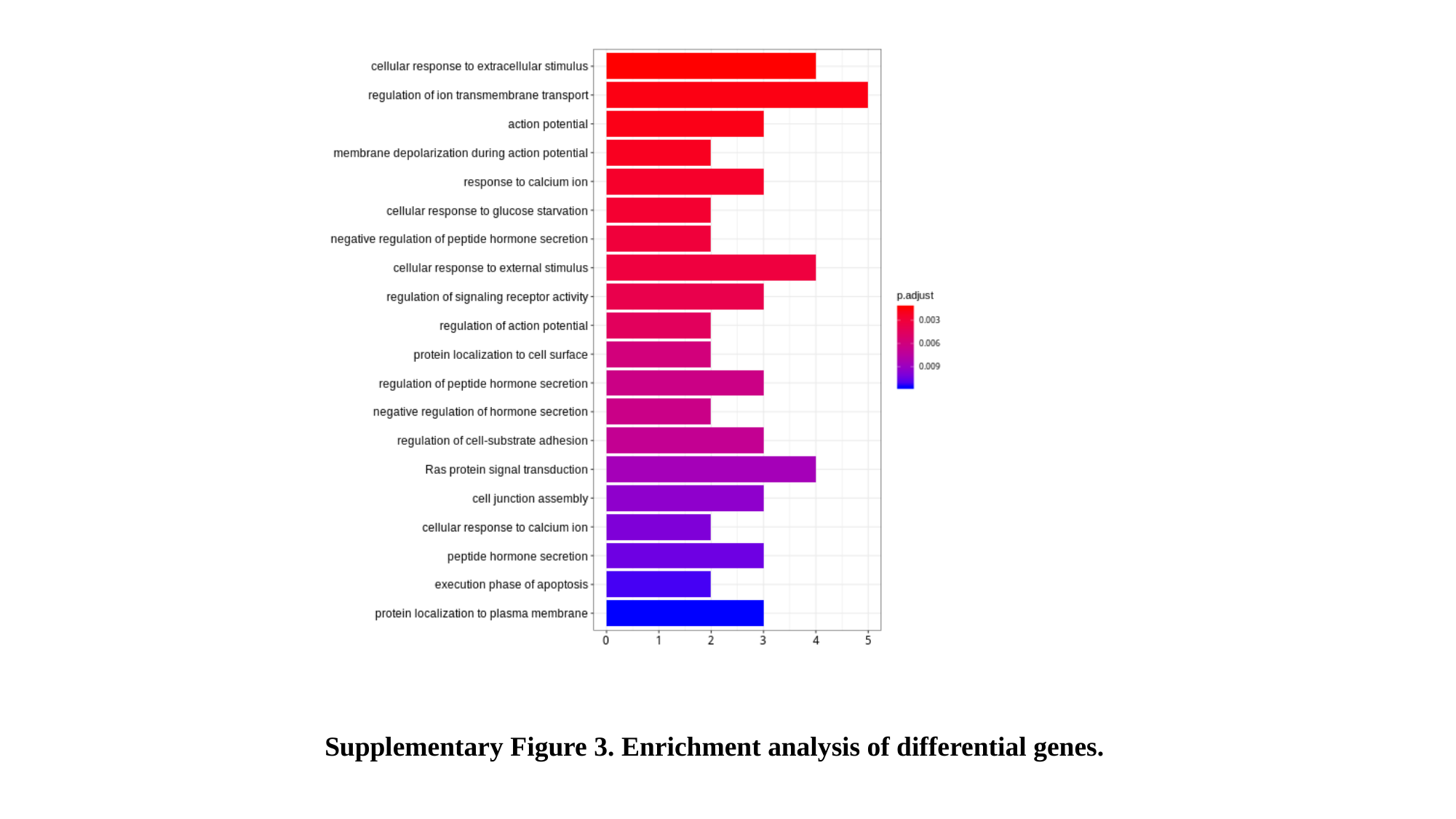

Supplementary Figure 3. Enrichment analysis of differential genes.

## Slide 4
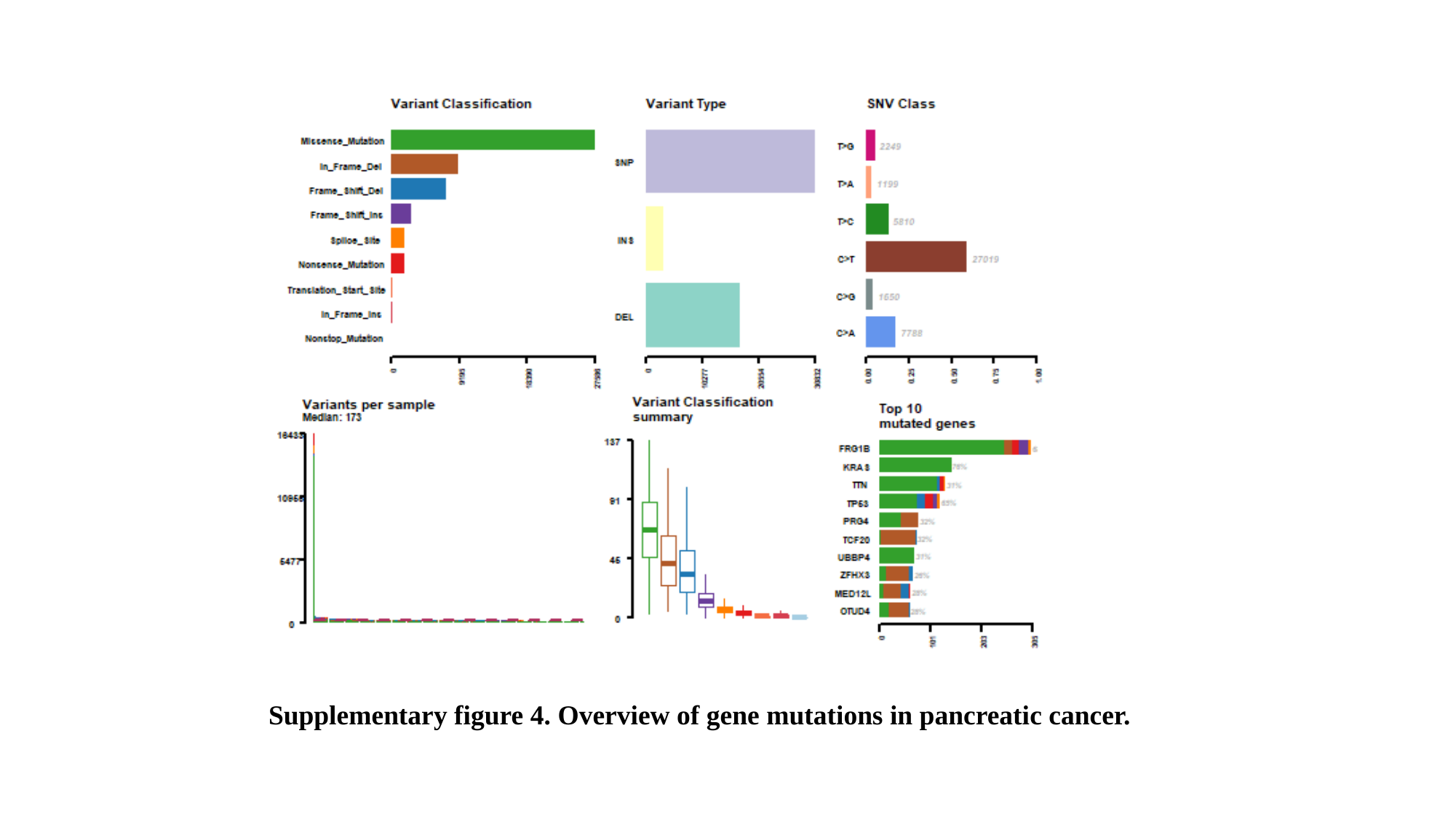

Supplementary figure 4. Overview of gene mutations in pancreatic cancer.

## Slide 5
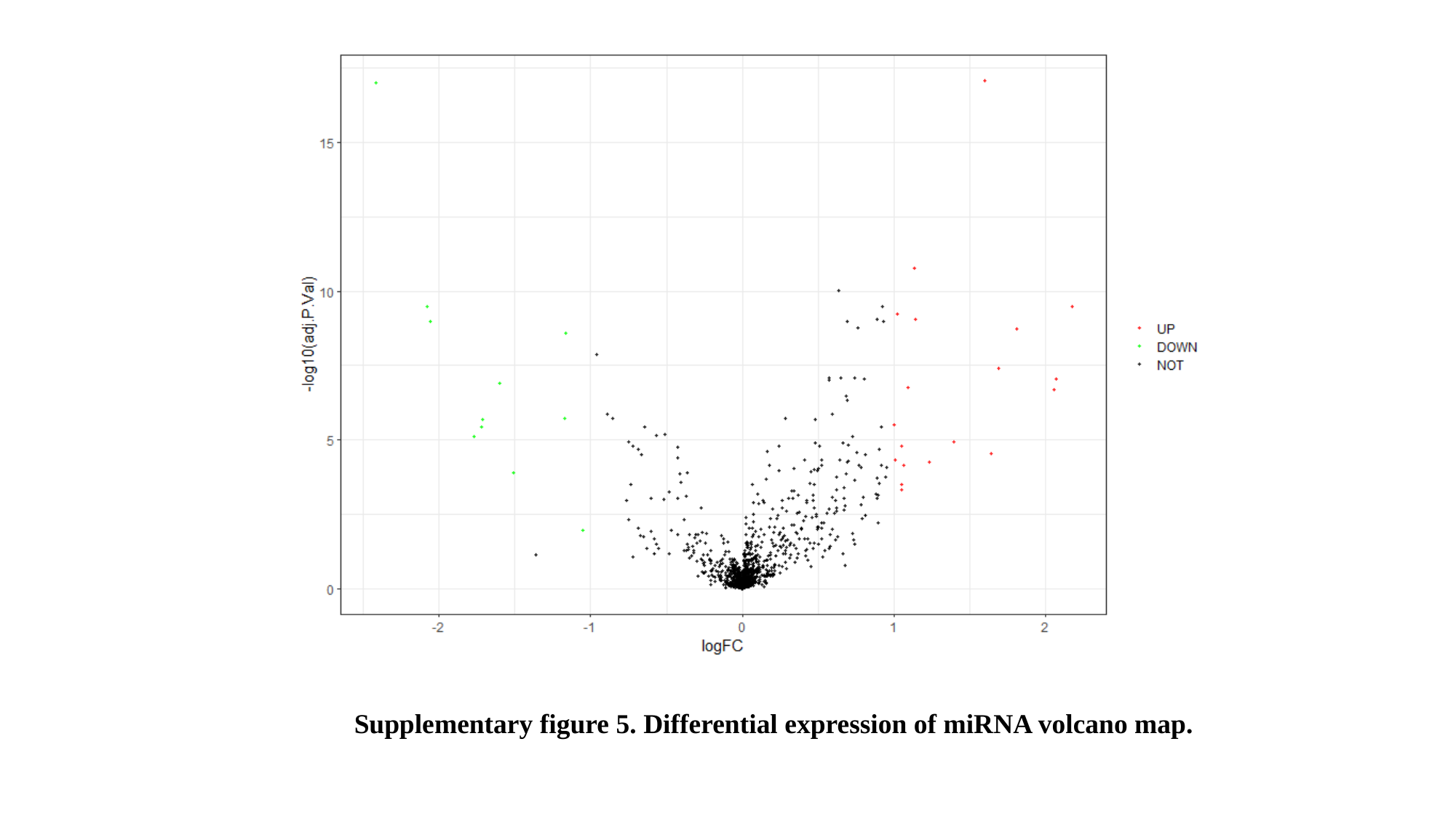

Supplementary figure 5. Differential expression of miRNA volcano map.

## Slide 6
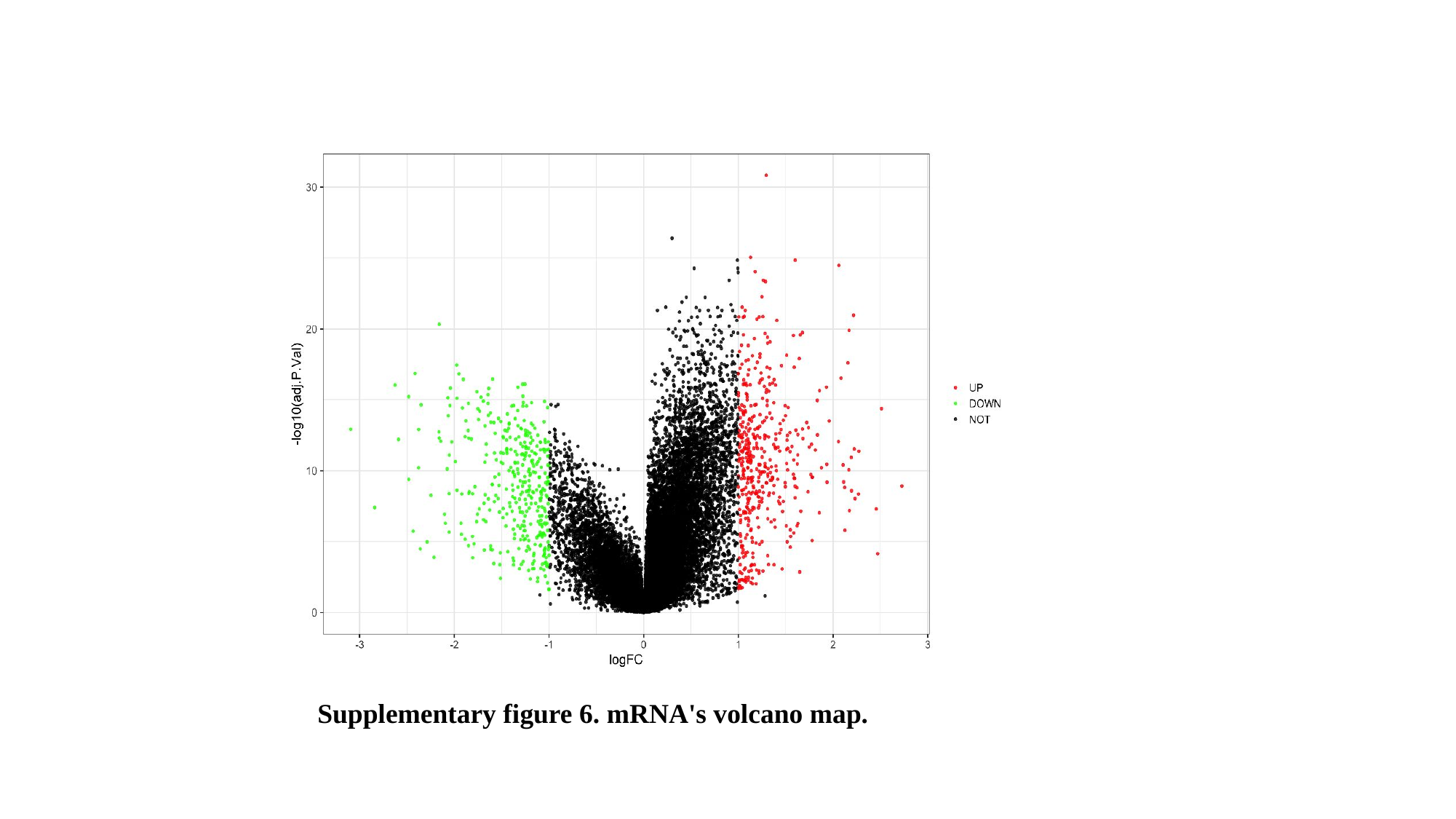

Supplementary figure 6. mRNA's volcano map.

## Slide 7
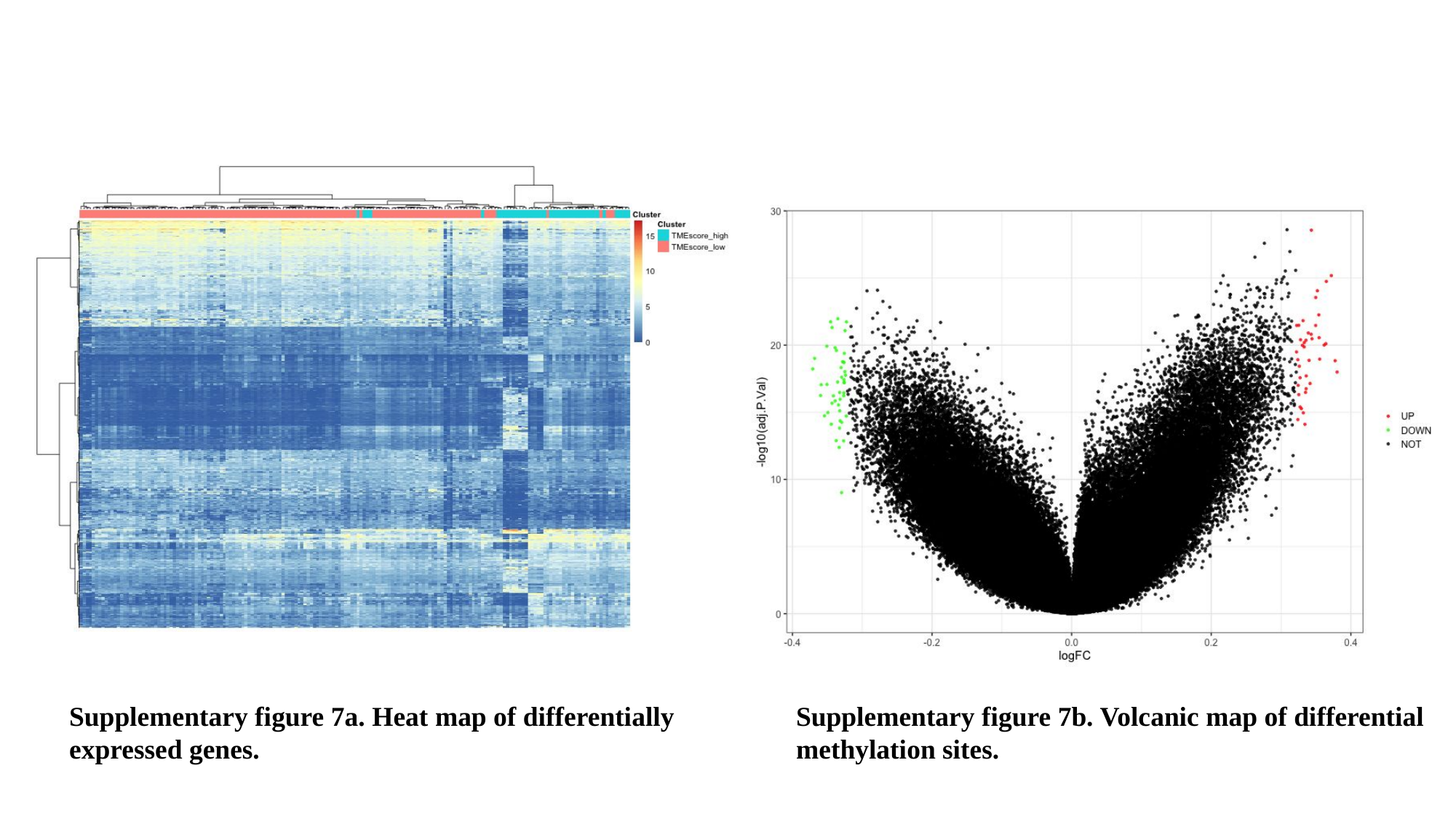

Supplementary figure 7a. Heat map of differentially expressed genes.
Supplementary figure 7b. Volcanic map of differential methylation sites.
